# Assessing social quality of sheltered independent housing: challenges of scale and group mix

**DOI:** 10.1007/s10901-017-9567-0

**Published:** 2017-08-30

**Authors:** T. G. M. Spierings, P. M. Ache

**Affiliations:** 1 Institute of Built Environment, HAN University of Applied Sciences, Deken de Louwstraat 11, 5401 BE Uden, The Netherlands; 2 Institute for Management Research, Nijmegen, Radboud University, Postbus 9108, 6500 HK Nijmegen, The Netherlands

**Keywords:** Group mix, (Physical) scale, Sheltered independent housing, Sheltered independent living, Social quality of housing

## Abstract

Forecasts for many cities and regions in Europe predict a growing share of ‘elderly’ people in the overall population. In addition to this general ageing process, the number of very old people is of specific importance for the issue under discussion. This article looks at sheltered independent housing and living. In particular, the article presents the results of a quantitative and qualitative multidisciplinary study of those facilities in the Netherlands. The research provides insight into the effect of physical scale and group mix on the social quality of sheltered independent housing. The results are based on a desk study of 265 projects and a detailed case study of 24 projects. The quantitative desk study provides reviews related to the time and location (and vice versa) and thus develops a picture of the variation in sheltered independent housing complexes for the period 1998–2010. The findings of the qualitative section in general are that ensuring security and belonging is an important function of sheltered independent housing for residents. Regarding the dimension of physical scale, the responses regarding the desired scale are surprising, with equal support for large as well as small scale. Preferences are strongly related to the location in towns or villages, as the scale surrounding the housing. Regarding group mix, the most important finding is a limit of tolerance between groups, particularly tolerance among vital elderly people towards groups of residents with a mental disability or dementia. This limit seems to be reached much sooner than commonly thought, or deployed on the basis of idealistic motives.

## Introduction

The ageing society is a standard in discussions about demographic development and the effect it has on both the changing demand side as well as supply side of our built environments. Forecasts for many cities and regions in Europe predict a growing share of ‘elderly’ people in the overall population (EC and DG Regional Policy 2011 October, p. 15):

‘The number of people aged 60 and above in the EU is increasing by more than 2 million every year, roughly twice the rate observed until about three years ago. By 2014, the working-age population of 20–64-year-olds is projected to start declining. As fertility remains considerably below replacement rates, in most EU Member States the relatively small EU population growth still observed is mainly caused by migration inflows. However, a detailed analysis at regional level reveals a more diverse picture of demographic patterns’.

In addition to a general ageing process, the number of very old people is of specific importance for the issue under discussion. ‘A dramatic increase in very old people is an important aspect of the ageing population. The number of those aged 80 and above will sharply increase, doubling every 25 years. In the next 30 years, this age group will represent more than 10% of the population in many EU cities’ (EC and DG Regional Policy, 2011 October, p. 17). However, the distribution across parts of the territorial and spatial structure varies. Some larger cities will continue to be ‘younger’ in relative terms; London and Paris are two examples. The countryside, in general, suffers most from ageing, with very remote places, for instance in Scandinavia, literally dying out. The costs of ageing are extremely large, in Germany, for example, almost EUR 40 billion will have to be invested in measures for structural adaptations (removal of obstacles in a flat, improvement of accessibility), with additional expenses of EUR 18 billion for age-adapted living standards (EC and DG Regional Policy 2011, p. 18).

That has effects. In addition to differences in the dynamics of the processes, varying between spatial categories, we will also see differences in the provision of services, in terms of formats or frequency and, especially, in the health sector. It is in this sector that we also find the object of interest in this article: sheltered independent housing for people who are old and in need of various levels of health-related support. This article looks at sheltered independent housing and living. In particular, the article presents the results of a quantitative and qualitative study of those facilities in the Netherlands. With that, we can fill a gap in the academic literature, as studies regarding the influence of physical scale on the social quality of sheltered independent housing are scarce.

The multidisciplinary research provides insight into the effect of physical scale on the social quality of sheltered independent housing. It consists of a desk study of 265 projects and a detailed case study of 24 projects. We also designed an online atlas to make the conclusions and recommendations available for decision-making for new initiatives (Spierings 2014). Overall, the aim is to contribute to a more informed and evidence-based assessment and discussion of sheltered independent living. After a conceptual section that explains some of the relevant concepts of our analysis, the article continues by presenting central empirical findings regarding two of the important dimensions, which are the scale and the social quality of sheltered independent housing and living.

At the end, the article discusses the implications of these findings for the design and organisation of sheltered independent housing and living facilities. An ageing society as such might be fit for the future, as health and nutrition trends in Europe point in that direction. The ‘grey’ is not just ‘gold’ but also active, placing different demands on housing and urban environments. However, diseases create a parallel phenomenon, with more people in need of care, especially those with Alzheimer’s disease, demanding new standards for living at home, namely assisted living at home. These developments come together in cities of all categories and sizes, where we need to create innovative solutions for an ageing society, from intergenerational co-housing (Ache and Fedrowitz 2012) to sheltered independent housing for people in need of care (Spierings 2014).

## The concept of sheltered independent housing and living

**Fig. 1:**
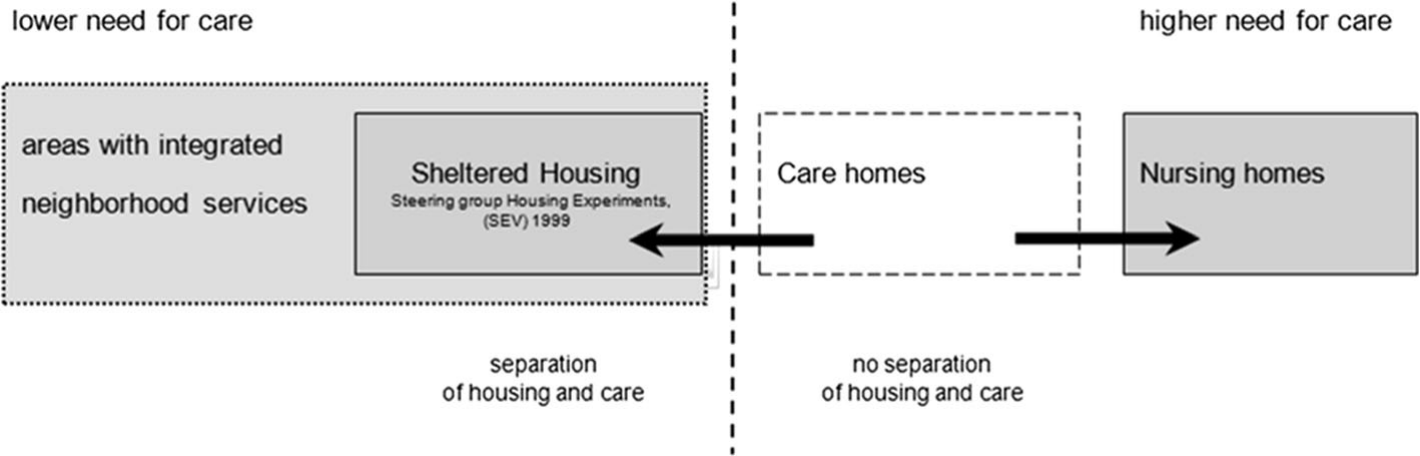
Transition in sheltered housing. *Source*: Spierings (2014)

Housing for the elderly in the Netherlands has been changing constantly since the 1950s. Once-valued homes for the elderly—like the *Huis in de Duinen, established in Zandvoort in 1955, a large scale, Non*-*Mixed building with central facilities* and *standardized rooms of 15* *m* ^*2*^ *; the project is a representative example of the reconstruction period after World War II when a new social security system was introduced* (Mens and Wagenaar 2009)—have been replaced during the so-called Golden Years in housing for the elderly in the 1960s and the large-scale housing explosion of the 1970s, by care homes and nursing homes (van der Voordt and Terpstra 1995), still large scale and Non-Mixed but with standardised rooms of up to 40 m^2^. After a period of smaller-scale urban infill and tailored work, partly guided by the depression in the 1980s, in the 1990s these homes were subsequently replaced by small-scale housing facilities (Boekhorst et al. 2008) for groups of six to eight elderly with dementia, with a private bedroom and bathroom, and a larger common living room. Residents of care homes are now housed in sheltered independent housing, scaled from 30 up to 300 elderly in apartments of 75 m^2^, mixed with elderly people with dementia or, more preferably, age at home, living in areas with integrated neighbourhood services (Edwards 2001; see Fig. 1) where housing, care and welfare are integrated in a neighbourhood of 10,000 inhabitants, like, for example, Prinsebeek in Breda (SEV 2012). The preferred goal can be formulated as: living independently for longer periods. The resulting question is: on which scale and in which group mix should we build and house our elderly to achieve not only longer independence but also the highest social quality?

Sheltered independent housing in the Netherlands has been an object of research for a number of years (Singelenberg 2005; Spierings 2014), and opinions on sheltered independent housing differ. The academic discussion considers sheltered independent housing facilities out of date as a form of housing and also lost the research interest in the subject. However, sheltered independent housing is still being built, changing in character and intended for a wider variety of target groups, like people with dementia and physical or mental limitations. The combination of different groups results in what can be called lighter or heavier versions of the concept (Singelenberg and van Triest 2009), with the level of care that is provided in a facility defining the scale of the sheltered independent housing, and vice versa.

Further than that, a systematic analysis of the physical scale of sheltered independent housing and its effect on the social quality of housing is rarely or not explored, neither in the Dutch context nor internationally. Initiators, therefore, decide on the basis of previous experiences, intuition and good intentions, at times guided by policy norms and always focused on business operations. However, with more recent initiatives in the Netherlands, in which a number of target groups are deliberately mixed and facilities are strongly developed, decision-makers aim to improve the social quality of housing and improve integration, again without any systematic research as backup.

**Fig. 2:**
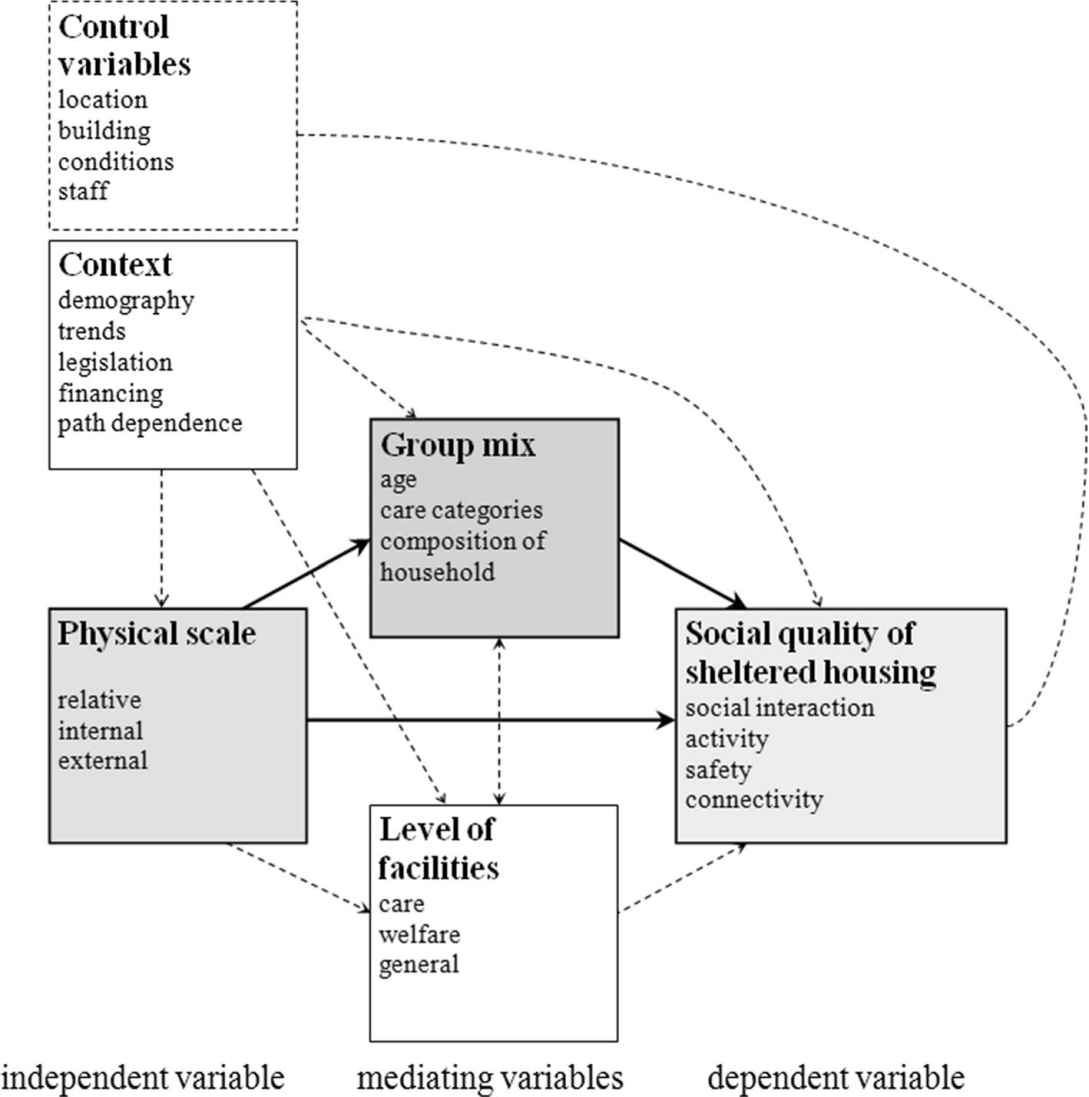
Conceptual model. *Source*: Spierings (2014)

For the current article, we focus specifically on the question whether a wider group mix of various care-needing residents leads to greater integration and a better social quality of housing (see Fig. 2). As already stated, research is scant, but when looking at the few available studies, we can see that small-scale living facilities for people with dementia have been researched within the field (van Liempd et al. 2010; Singelenberg and van Triest 2009; te Boekhorst 2007). The positive side of findings led to a revaluation of small-scale housing in the policy of the Dutch government (Bussemaker 2009). However, objections arose as well (Geelen 2005; Kiers 2007). Groups are sometimes too small to allow for an individual to find a person that affects him or her; personnel is on its own, which leads to inflexibility and reduced pleasure in work.

The current article and research attempts to fill that gap. The conceptual relations between physical scale and group mix, and their combined effects on the social quality of sheltered housing, are outlined in Fig. 2. The shaded boxes in Fig. 2 depict the core cause-and-effect relations that guided the research and empirical work.

We have to acknowledge the impact of social and political changes since the 1990s, since large and successive changes in legislation as well as in funding of housing and care in the period 1998–2010 influenced these variables.

The separation of the financing of housing and care in the Exceptional Medical Expenses Act (AWBZ) between 1995 and 2009 and the introduction of the Wet Maatschappelijke Ondersteuning in 2007 (Social Support Act, WMO), regulating the budgetary role of local communities in care and welfare, had further impact on the provision of care and facilities. Concerning the licensing system in building for residential care, another major change took place in 2008 when the Netherlands Board for Healthcare Institutions (CBZ) lost its licensing authority. All of these developments, together with the financial crisis, led to a situation of great uncertainty in the financing of housing and care projects, and therefore to major stagnation, and had an adverse effect on experiments in the development of new projects.

### Definition of scale

A central concept in this article is scale, a dimension that obviously shows variations. We approached the scale concept from two distinct angles, combining organisational theory with architectural theory. Organisational theory is relevant, as we formulate the hypothesis that sheltered independent housing and living can be seen as an organisational challenge in addition to a challenge of finding the right building scale.

The literature study provided a number of dimensions, which help further differentiate the scale dimension. Management theory, especially introduced by De Groot (van Zijp 1997), studies three concepts of scale: the physical scale, the structural scale and the mental scale. The physical scale refers to the number of social and spatial units. In the current article, physical scale concerns the number of homes in a project. The structural scale refers to the scale of the organisation and in particular to process, in this case the care and service provision. Finally, the mental scale refers to experienced cultural patterns. This can be found both within a group and in the emotional bond of a group and relates mostly to the inclusion or exclusion of different target groups. Of these three concepts, physical scale is the independent variable, whereas structural scale and mental scale are perceived as intermediary variables that are affected by the physical scale and affect the social quality of housing (see also Fig. 2).

In architectural theory, physical scale is an obvious focus. The architectural theorist Boudon (1978) defines scale as the ratio between a building and a single element, for instance an entrance, and the architectural concept of proportion as the mathematical expression of the ratio between elements like different entrances. Ching (1979) states that scale alludes to the size of a reference, like the size of a person, in relation to the size of an entrance. He defines generic scale as the size of an element in comparison with the size of other elements in the same context. In line with these theories, three concepts have been defined for our research with regard to the measurement of physical scale: the external, the relative and the internal scale. The external scale, comparable with the generic scale of Ching, refers to the size of the service area of the assisted living facility. The relative scale is the size in comparison with other projects. And finally, the internal scale, similar to proportion in the concept of Boudon, is the partition with respect to internal groups.

### Definition of group mix

The second important dimension of sheltered independent housing and living facilities, in addition to scale, is group mix. Sheltered independent housing and living facilities are usually built for certain groups of ‘customers’. Several existing group classifications were examined to systematically compare shared housing and living facilities and to identify relevant case studies (see also Table 1). They come from various sources, like legal acts or care providers.

**Table 1:** Group mix. *Source*: Spierings (2014)

Groups					SET		
	Exceptional Medical Expenses Act AWBZ legislation	Profiles TNO	Database Expertise Centre Housing and Care KCWZ	Groups in this research	1	2	3
Vital elderly		Elderly with few or no limitations	Elderly	55 + with no or modest care need	x	x	x
Care groups	Psycho geriatric patients	Dementia	People with dementia	People with dementia		x	x
	Mentally handicapped		People with a mental handicap	People with a mental limitation		x	x
	Psychically handicapped; Sensory handicapped; Somatic patients	Elderly with large physical limitations; Elderly with mobility and personal care limitations; Elderly with mobility limitations	People with a physical handicap	People with a physical limitation		x	x
	Psychiatric patients		People with psychiatric problems	People with psychiatric problems		x	x
Non-care groups			All (other) neighbourhood inhabitants	Families			x
				Starters			x
				Juniors			x

The Algemene Wet Bijzondere Ziektekosten (*Exceptional Medical Expenses Act*, AWBZ) in the Netherlands operates on the basis of six different care groups, defined in terms of care requirements. In addition, the Netherlands Organisation for Applied Scientific Research (TNO) developed a method related to the profiles for care requirements (TNO 2010). The Expertise Centre Housing and Care (KCWZ) uses a broader classification and comes to the following distinct groups: people with dementia; people with physical disabilities; people with mental disabilities; people with psychiatric problems; the elderly; and all other residents (KCWZ 2010).

On the basis of the above categories, the choice of cases for our study was determined, dividing the selection according to group mix. Three sets of cases were defined (see Table 1, Columns 1–3): The first set is characterised by the ‘Non-Mixed’ of the first generation of sheltered independent living units (SET 1). The second set is that of complexes ‘Mixed with Heavier Care’ (SET 2), and the third set is ‘Mixed with Heavier Care and No Care’ (SET 3).

### Definition of social quality of sheltered independent housing

Finally, the dependent variable social quality of sheltered independent housing is the core interest of this research. To begin with, the social quality of sheltered independent housing can be addressed by using existing definitions of quality in general and quality of sheltered independent housing in particular. Regarding general definition of quality, van der Voordt (2009) refers to a widely used definition of quality as the extent to which a product fulfils the requirements set for it. In architectural theory, Alexander (1979) defines a ‘central quality’ in each city or building, which is on the one hand objective and precise, but on the other hand not exact at all (liveliness, flexibility, wholeness, comfort and safety). Zwart (1989), from the theory of housing ecology, distinguishes building quality and the quality of housing and breaks them down into technical and economical quality, on the one hand, and functional, social, psychological and cultural quality, on the other hand. Finally and again from architectural theory, de Vreeze (1987) defines social, aesthetic and technical quality, which is very much in line with the Vitruvius concept of utility (Utilitas), beauty (Venustas) and reliability (Firmitas).

In addition to before-mentioned general aspects, for this research we use the definition of the social quality of housing from the memorandum People, Demands, Housing VROM (2000): the extent to which housing and the environment contribute to the social participation of citizens. It concerns increasing independence, choice and demand by citizens who need care or supervision and increase the opportunities to live independently for longer. At home environment involves increasing the satisfaction of residents with their neighbourhood and district, feel at home, vitality networking, solidarity, sense of responsibility for the environment.

This definition is in line with the psychological, social and cultural indicators for quality of housing from the authors above and consistent with the four indicators used for this study, all of them as perceived by the residents: the social interaction among residents and groups, meaning the amount of contact between people individually and the groups; the variety of activities, meaning the perceived amount and variations within the sheltered independent housing project that gives vibrancy and viability; the safety, meaning feeling at home and feeling safe which results in social control or anonymity; and the connectedness, meaning the individual together with other residents as one group resulting in familiarity and identifiability.

## Methods

The empirical research for our study consists of two parts: quantitative desk research into 265 projects and qualitative multiple case study of 24 projects.

### Desk research

The desk research on the basis of the archive of the Netherlands Board for Healthcare Institutions CBZ (CBZ 2001–2007) and Expertise Centre Housing and Care database (KCWZ 2010) presents the relationships between physical scale, group mix and level of facilities, and the relationship with legislation and funding during the period under consideration. Both databases are controlled, filtered according to the research question and analysed for associations and significance of correlations. In this article, we focus on the results from the KCWZ database, which is the most comprehensive database.

### Multiple case study

In addition to the desk research and statistical analysis of sheltered independent housing, a study of 24 cases was conducted. The distribution and variation of sheltered independent housing was the prime consideration in the strategic selection of the cases. For this selection, the KCWZ database was taken as the basis on account of the higher representative nature of this database for sheltered independent housing, the larger time span, and the completeness of the data. What is also reflected in the qualitative case studies is the overrepresentation of projects accommodating Mixed Groups With Heavier Care, which we found in the desk research. The tendency of mixing different groups, which can be seen as a result of the outlined changes in legislation and funding regimes, has been further investigated in the interviews with decision-makers. In addition, as an extra case, we studied Mixed Groups with Heavier Care and Non-Care, as an outlier identified in the desk research.

The multiple case study presents the influence of variation found in physical scale, group mix, level of facilities and the experience of social quality of living on the basis of a strategic selection from the desk research of 24 cases. Semi-structured interviews were conducted in 2011 with 174 residents, 40 professionals and 35 decision-making employees of sheltered independent housing complexes, applying an intensive narrative research method (Van Biene et al. 2008; Jansen et al. 2013). The narratives are arranged in sets of cases according to the research variables of physical scale, group mix and amenity level, in order to conduct not only a qualitative but also a quantitative analysis (according to the qualitative comparative analysis method, see Ragin and Rihoux 2008; Wagemann and Schneider 2010). The broad narrative analysis has delivered a very large amount of data, providing not only rich content but also complex information. The interviews were all transcribed and coded with 50 codes in the softwaretool Atlas.ti regarding What, How and Who, according to the method van Van Biene et al. (2008). Queries were conducted for all interrelated research variables from the conceptual model (see Fig. 2) in addition to the positive and negative satisfaction.

## Findings

### Findings desk research of 265 sheltered independent housing projects

**Fig. 3:**
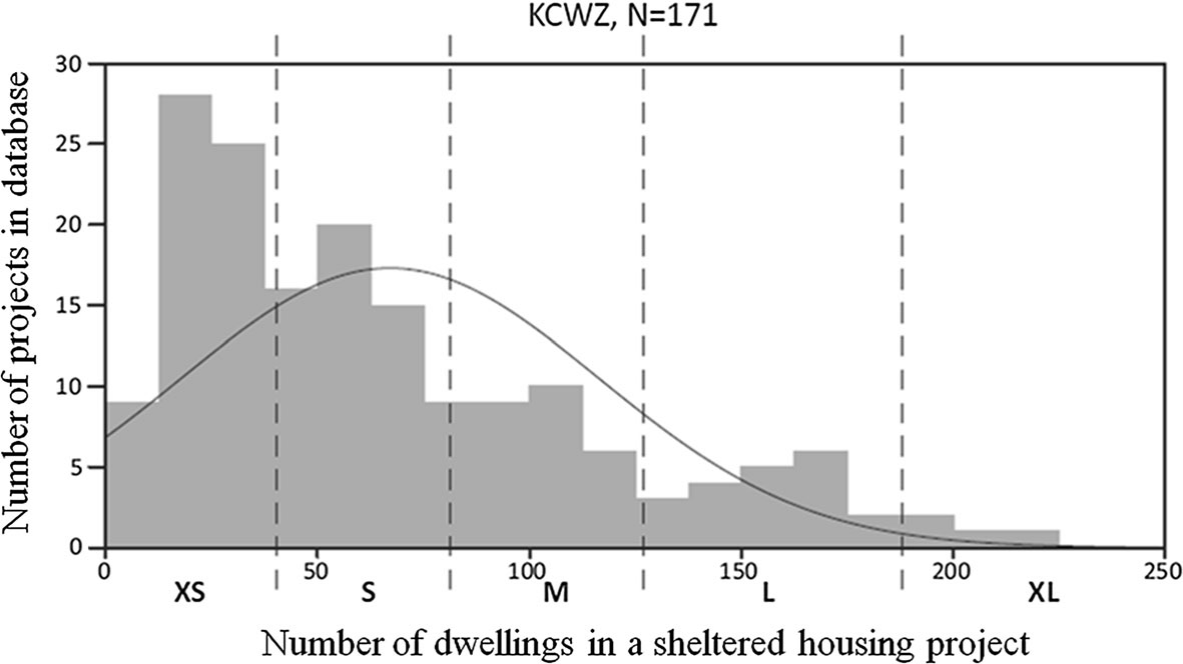
Range of physical scale and partition in physical scale groups. *Source*: KCWZ (2010) and Spierings (2014)

When we look at physical scale in the KCWZ database (Fig. 3), we see a range from 8 to 224 housing units per project. Clearly, there are more very small and small-scale projects than large-scale projects. The range in physical scale is used for classification and strategic selection of the case studies.

**Fig. 4:**
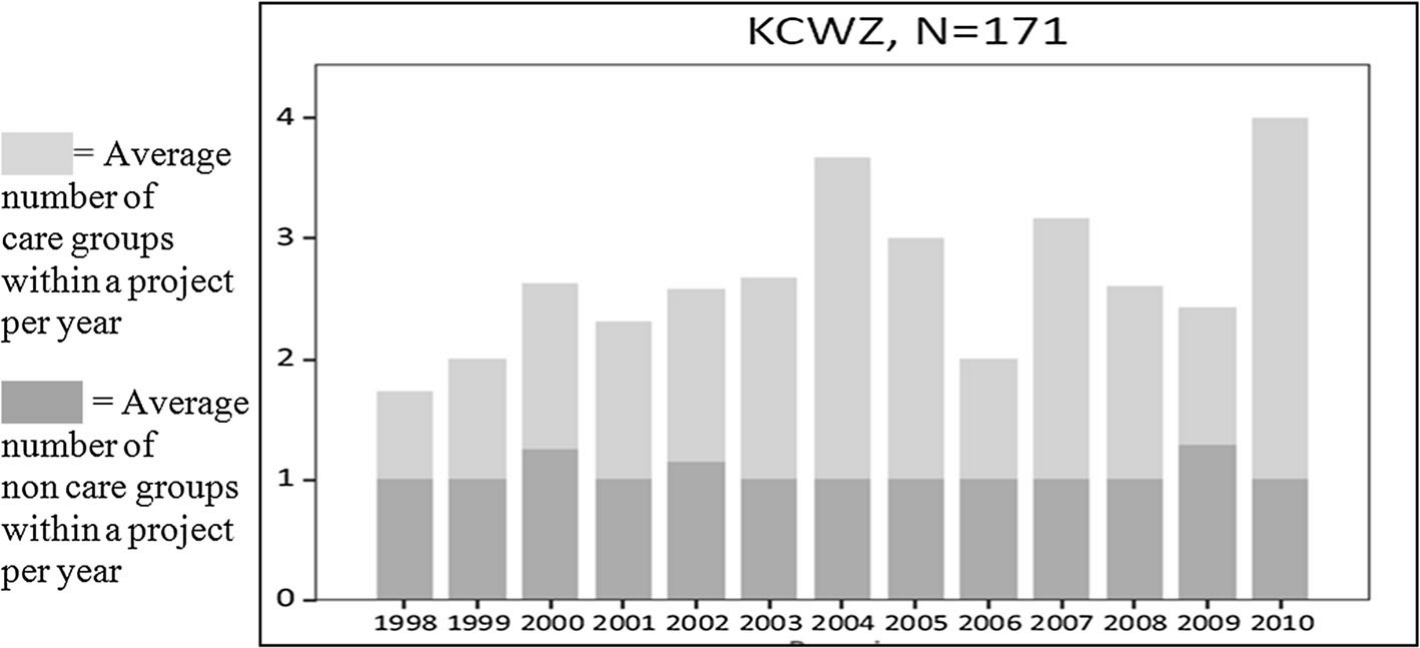
Group mix according to year built. *Source*: KCWZ (2010) and Spierings (2014)

When we look at group mix (Fig. 4), presented as the average number of non-care and care groups within a project over the research period, we can see over the years a very constant presence of one non-care group, which is plausibly the independently living elderly as defined in Table 1. The high numbers of care groups running from .8 to 3.0 per project reveal a striking overrepresentation of projects of Mixed with Heavier Care (SET 2 in Table 1). And looking at the small number of non-care groups larger than 1, we see a very low level of mixing with other non-care groups such as youngsters, families and starters (SET 3 of Table 1).

When we look at correlations between physical scale, group mix and changes in legislation (Table 2), we see a correlation between changes in legislation and funding and an increase in group mix. Therefore, we have focused on group mix during the qualitative case studies.

**Table 2:** Correlations between legislation period, physical scale, group mix (KCWZ) 2010. *Source*: Spierings (2014)

Spearman’s rho	Legislation period	Physical scale	Group mix
Legislation period			
Correlation coefficient	1000	.038	.243*
Sig. (2-tailed)		.622	.025
*N*	171	171	85
Physical scale			
Correlation coefficient	.038	1000	.211
Sig. (2-tailed)	.622		.052
*N*	171	171	85
Group mix			
Correlation coefficient	.243*	.211	1000
Sig. (2-tailed)	.025	.052	
*N*	85	85	85

* Correlation is significant on the .01 level (2 tailed)

In summary, the desk study provides quantitative reviews of this investigation and mediating variables related to time, location and interrelated and, thus, a picture of the variation in sheltered independent housing complexes in the period 1998–2010.

The observed variation in physical scale is used for classification in scale groups for strategic selection.

**Fig. 5:**
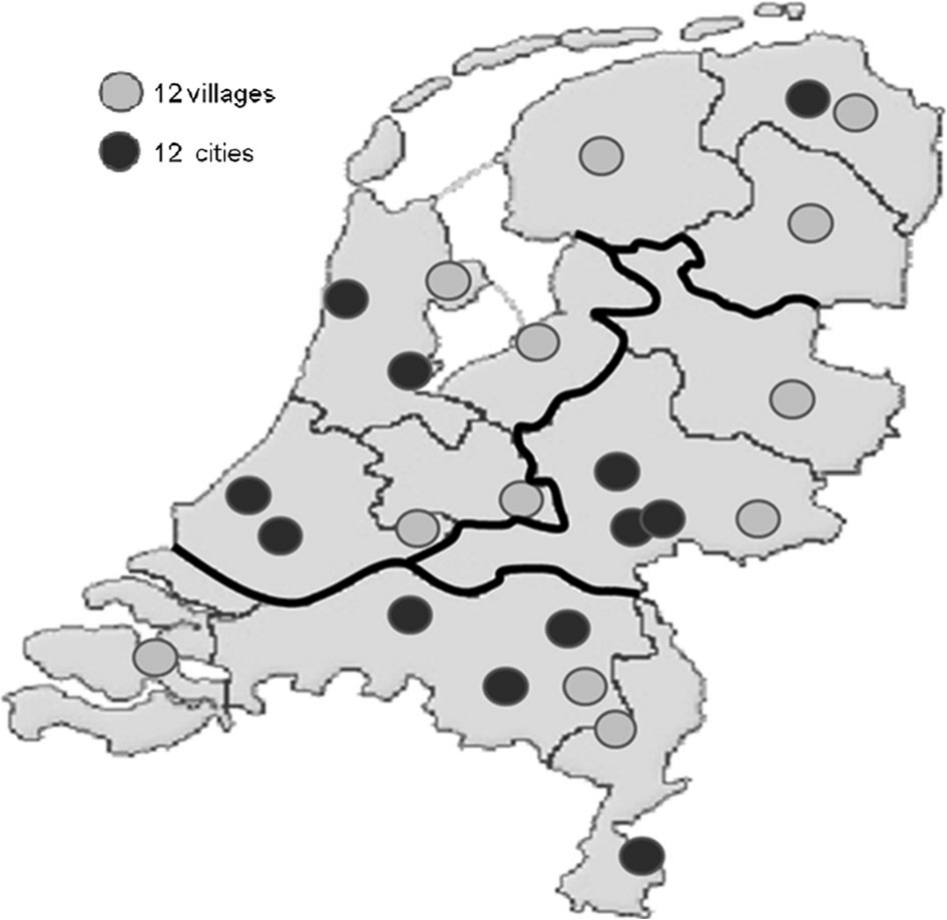
Map of locations for multiple case study (*N* = 24). *Source*: Spierings (2014)

There is a striking overrepresentation of Mixed with Heavier Care and an increase in the mixing and correlation with changes in legislation and funding (Fig. 5).

### Finding case study of 24 sheltered independent housing projects

**Fig. 6:**
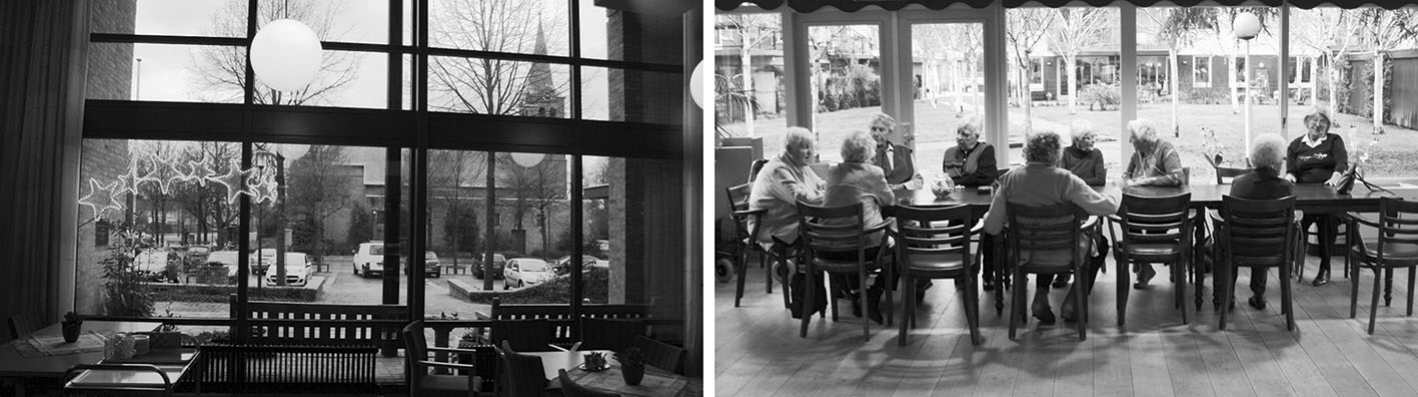
Variations in physical scale: extra small-scale case De Berken, Millheeze, and extra large-scale case Menno Simons, Amsterdam

**Fig. 7:**
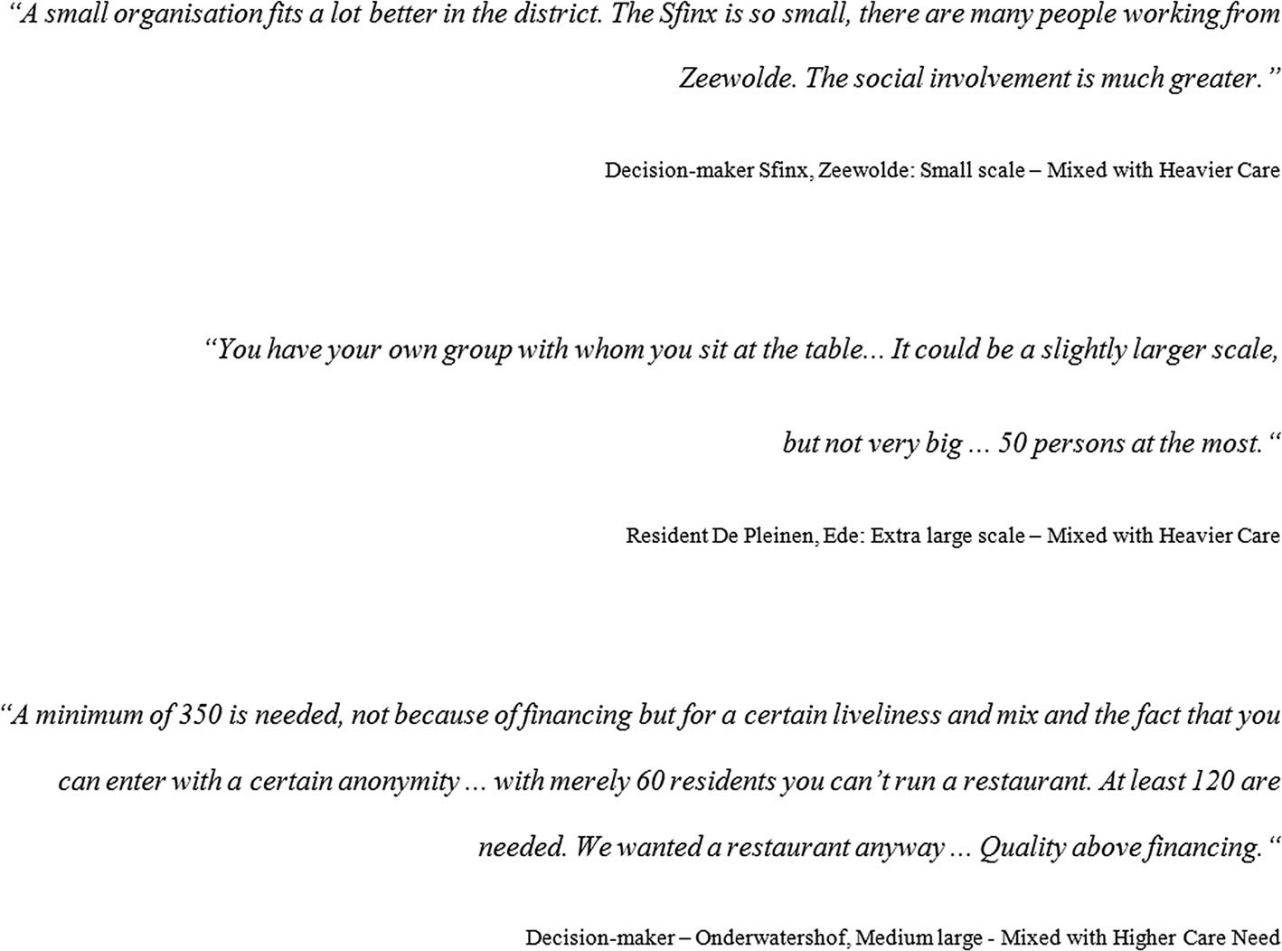
Quotes regarding physical scale from the case study (*N* = 24). *Source*: Spierings (2014)

Table 3 provides an overview of the strategic selection of the multiple case study for physical scale and group mix. The strategic selection also produced a representative range of physical scale (see Fig. 6). The spread of the group mix has an overrepresentation of cases of Mixed with Heavier Care and a single case of Mixed with Heavier Care and Non-Care (Malburgstaete, Arnhem, see Fig. 7). This is in line with the representation of the population in the desk research.

**Table 3:** Overview variables multiple case study (*N* = 24). *Source*: Spierings (2014)

Physical scale/group mix	(extra) Small <80	Medium 81–130	(extra) Large >131
Non-Mixed SET 1	De Wemel Wemeldinge	Jean Sibelius Eindhoven De Schermerij Leersum	Absent
Mixed with Heavier Care SET 2	De Sfinx Zeewolde Eilandstaete Arnhem De Berken Millheeze Domus Bona V Nederweert Huize St. Franciscus Veendam Nij Dekama Weidum	Rigtershof Grootebroek Onderwatershof Rijswijk BaLaDe Waalwijk ‘t Derkshoes Westerbork Het Reggedal Enter Het Spijk Eefde St. Annahof Uden	Bergweg Rotterdam De Pleinen Ede Reinaldahuis Haarlem Parc Imstenrade Heerlen Menno Simons Amsterdam Mercator Groningen Huis ter Leede Leerdam
Mixed with Heavier Care and No Care SET 3	Absent	Absent	Malburgstaete Arnhem

To reach saturation of results in a narrative study, Robson (2002a, b) defines theoretical saturation with 30 respondents. With an average number of eight residents interviewed in each case, eight cases are also needed to achieve a theoretical saturation in sets. This theoretical saturation is successfully fulfilled in the sets regarding physical scale. But here, the theoretical saturation in two of the three sets regarding group mix was not successful. Nevertheless, the sets were thoroughly investigated; for SET 2, we conducted 3 case studies with 24 respondents, giving a plausible practical saturation, and for SET 3 a single case was conducted.

Eight residents, one or two decision-makers, and one or two professionals were interviewed in each of the 24 cases. For the testing of the hypotheses, the cases were arranged in sets (see also Table 1), varying according to the three independent and mediating variables. These sets were quantitatively and qualitatively analysed in a combination of qualitative comparative analysis (QCA) (Ragin and Rihoux 2008; Wagemann and Schneider 2010) and a narrative method (Van Biene et al. 2008; Jansen et al. 2013).

Cases are divided equally between villages and cities and proportionally spread over the Dutch regions and provinces (see Fig. 5). Sixteen of them were newly built, six were partly extended, and two were totally renovated. Within the most represented SET 2, Mixed with Heavier Care, two specific cases can be identified: St. Annahof Uden, where the welfare component is fully organised by the residents themselves, and Parc Imstenrade, Heerlen, the single case run by a commercial party and not a housing association in combination with a care organisation (*).

### Focusing on the narrative analysis

The broad narrative analysis of 174 residents, 40 professionals and 35 decision-makers has delivered a very large amount of data, providing not only rich content but also complex information. The interviews were all transcribed and coded with 50 codes in the softwaretool Atlas.ti regarding What, How and Who, according to the method van Van Biene et al. (2008). Queries were conducted for all interrelated research variables from the conceptual model (see Fig. 2) in addition to the positive and negative satisfaction.

**Fig. 8:**
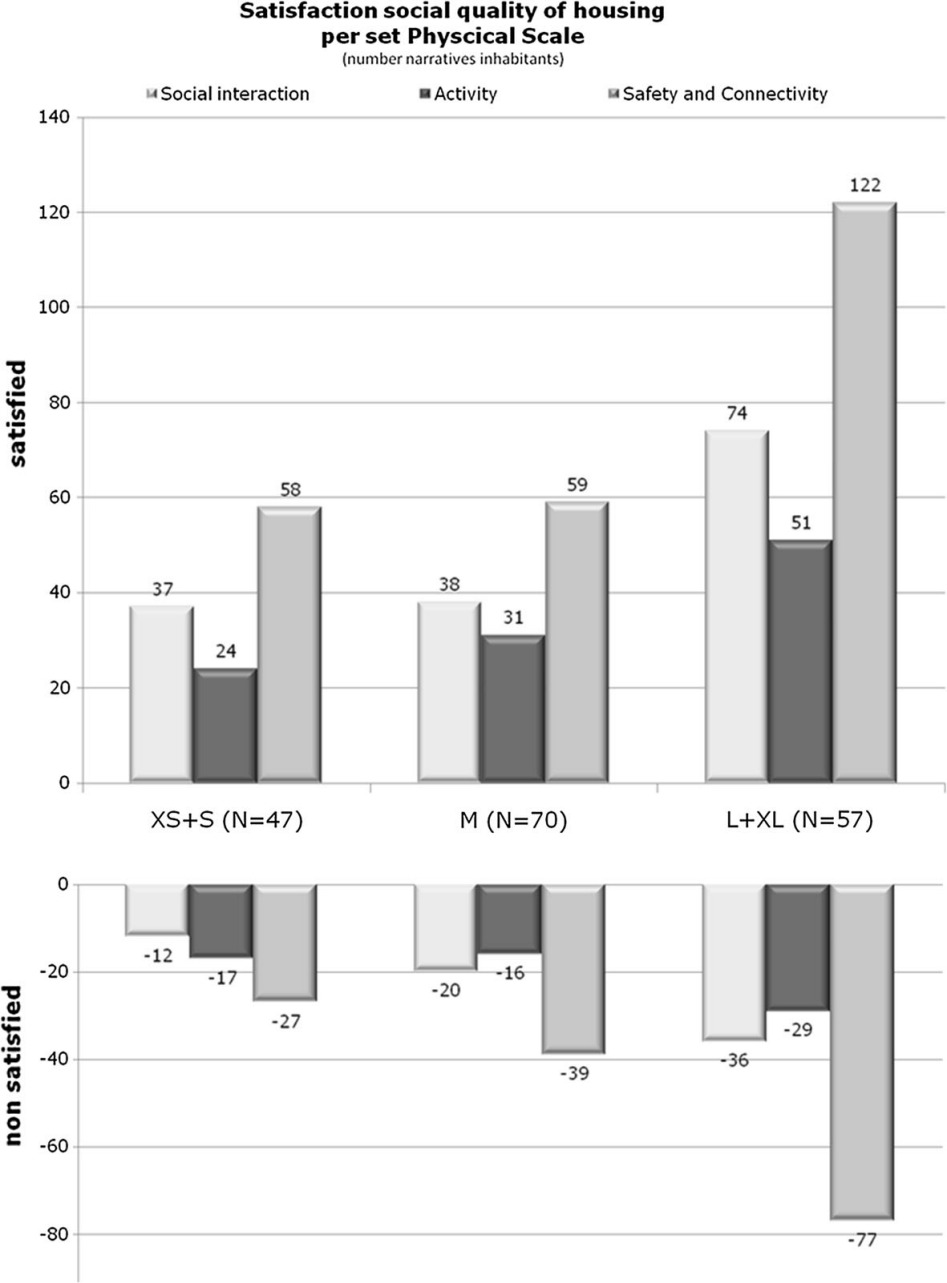
Satisfaction social quality of housing per set physical scale. *Source*: Spierings (2014)

The results were presented in quantitative overviews (see Fig. 8) and in qualitative overviews arranged in 23 themes with exemplary narratives per relation between research valuables and per respondent group.

The firmest result in general is that ensuring security and belonging is an important function of sheltered independent housing for residents.

Regarding the dimension of physical scale, the responses regarding the desired scale are surprising, with support for large scale as for small scale. Their preferences were strongly related to the location in towns or villages, as the external scale of their housing (see Fig. 7).

**Fig. 9:**
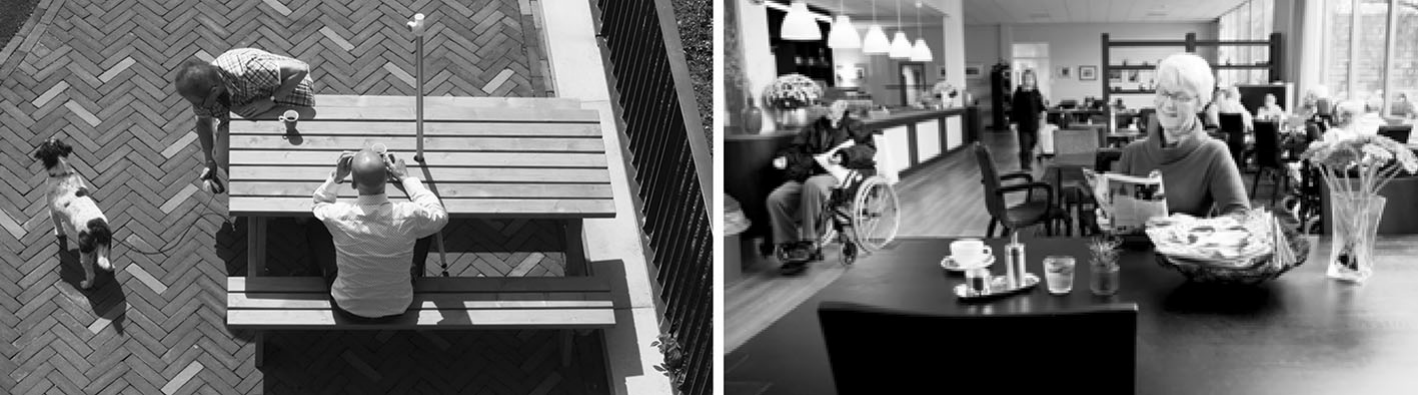
Variations in group mix: one of the three Non-Mixed cases Jean Sibelius, Eindhoven, and single case Malburgstaete, Arnhem, Mixed with Heavier Care and No Care project

**Fig. 10:**
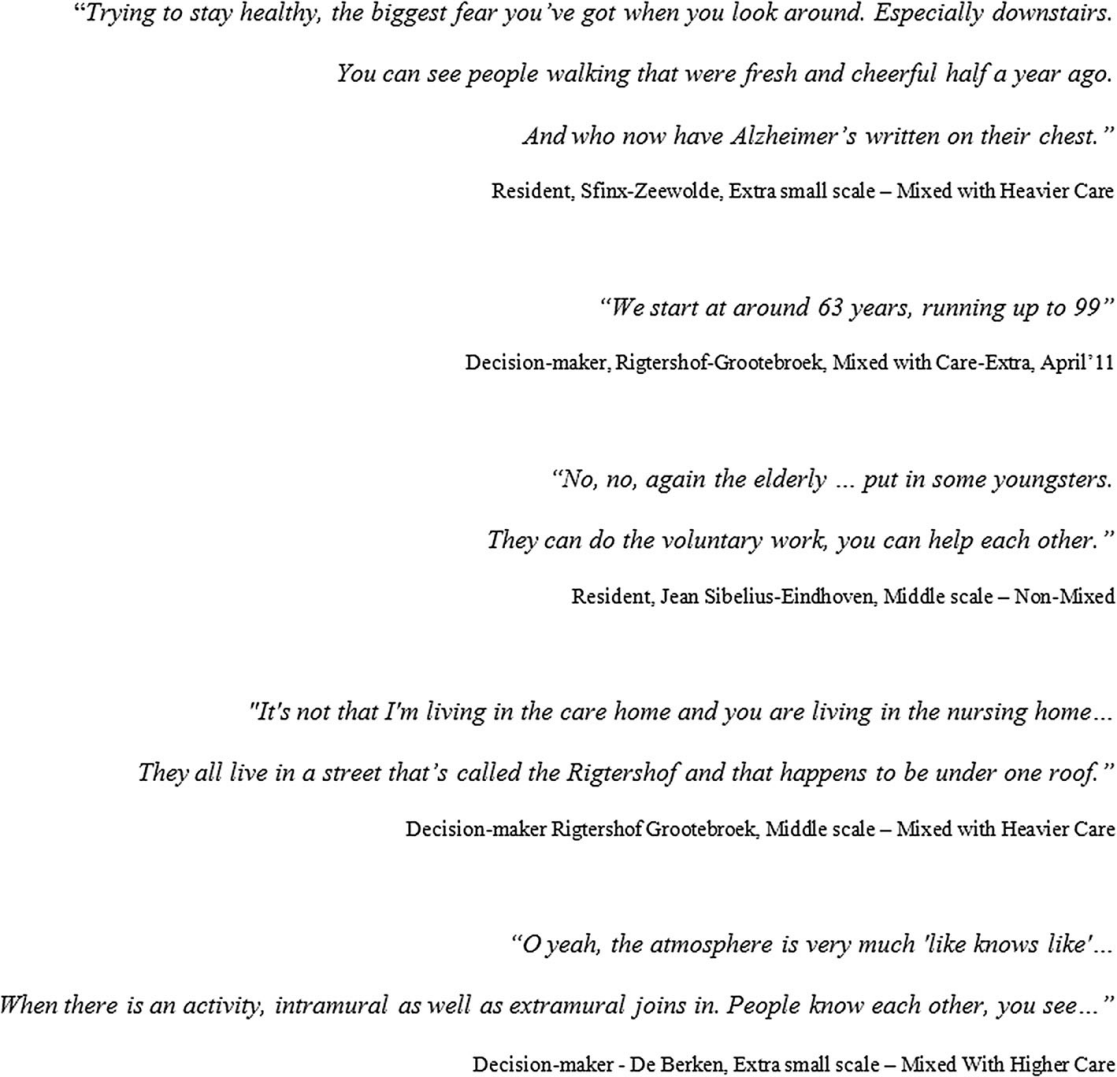
Quotes regarding group mix from the case study (*N* = 24). *Source*: Spierings (2014)

Regarding the aspect of group mix, the most important finding is a limit of tolerance between groups, particularly the tolerance among vital elderly people towards groups of residents with a mental disability or dementia (Fig. 9). This limit seems to be reached much sooner than commonly thought or deployed on the basis of idealistic motives for group mix (see Fig. 10).

The main interest of this research and article is to explore the relation between physical scale and group mix and the resulting effects on the social quality of sheltered independent housing. In particular, the perceived social interaction between residents and groups, the perceived variety of activities, the perceived safety and the perceived connectedness can be seen as composing the social quality of sheltered independent housing. Taking this as a lens for the reporting of additional results, we can present the following findings across both parts of the empirical research. This will be followed by a set of recommendations that we provide on the basis of our findings in each set of cases with different characteristics in scale and group mix.

#### Appreciation small and large scale

We started our article by discussing the various scale dimensions. The data show an even distribution of physical scale among sheltered independent housing in the Netherlands for the period 1998–2010, despite the preference of both the government and investors for either small or large scale. What also surprises is that there is no visible correlation between the size of sheltered independent housing and their location either in small villages or larger towns. Nor does the higher demand in cities lead to a wider diversification of sheltered independent housing. Small-scale facilities are appreciated for a number of reasons; residents and decision-makers value the expected domesticity, and safety decision-makers value the possibilities for customisation. Large-scale facilities are seen as positive because of their liveliness, choice of contacts, activities, but also their anonymity. Despite some cultural differences within the Netherlands, the desired scale and, related to that, the expected level of social quality of housing does not differ according to region in the Netherlands.

#### Limited informal care and large group mix

As was demonstrated, most groups in sheltered independent housing present a mix of different care needs. The proportion of sheltered independent housing with a wider group mix of residents increases. Legislation and funding regimes influence this dimension. That obviously results in some frictions, as groups with a lighter level of required care and fewer physical limitations can be better integrated compared with heavier cases of care. The size of the facility then makes a difference, because within larger facilities more diverse groups can be cared for more easily. Sheltered independent housing is based on a formalised provision of care, which undermines informal, mostly family provided care. Finally, a particular feature of group mix is the occurrence of relational aggression, which is more frequent in mixed groups and in larger facilities. That has an impact on the social quality of housing (feeling at home).

*In an ideal world, the customers of sheltered independent housing search for a situation in which they can live* independently but within a shelter of safety and comfort, in an environment of social interaction, created by living together in one building with a communal room and preferably a 24-h presence of care personnel. Our results show that the expected physical and health-related safety and social interaction are the main reasons to move into an Assisted Living Facility. Sheltered independent housing is a useful and valuable alternative to ageing in a less suitable home or neighbourhood, being forced to move into an intensive institutional care home, or living independently as an elderly or ageing person.

## Recommendations and reflections

And, finally, what recommendations can we make regarding the scale and group mix we should build to house our elderly and achieve the highest social quality? Given the expressed preferences of elderly people, there is no single optimal value for the physical scale. The empirical results reveal that there is no optimal value of physical scale and group mix. However, despite the non-existence of an ‘optimal’ physical scale, we still can use the findings to discuss the composition of individual sheltered independent housing and living facilities.

Judging from the analysis conducted, we conclude that an optimal size for vibrancy and viability, familiarity and identifiability, but also social control and anonymity, seems to be between 25 (sufficient vibrancy) and 120 U (social control). Familiarity and identifiability seem to have an upper limit of 300 U. This is reflected in the external scale, with 25–120 housing units a good match for village locations with fewer than 25,000 inhabitants; the range for urban locations with over 25,000 inhabitants falls between 80 and 350 U.

Regarding group mix in terms of required level of care, age and vulnerability, the aim should be to establish a balanced range of level of care needed. ‘Vital’ elderly (i.e. elderly people needing only light forms of care) should have a minimum share of 30%, which according to our findings would also be the maximum share for people in need of heavier care, such as those suffering from dementia or somatic. Regarding the combination of age and vulnerability, a share of 30% minimum seems to be reasonable.

In the discussion of the social quality of sheltered independent housing, a more diversified perspective of sheltered independent housing is certainly required. As will be discussed further down, variety and diversity are needed. Future changes in legislation must create flexibility in the legal and public administrative system, and the dimension of housing needs definitions. A very practical proposal, based on our findings, is to transform existing apartment complexes into sheltered independent housing complexes by adding a communal space and a 24-h care component.

A final recommendation relates to the governance and decision-making process. Sheltered independent housing should not just be left to the ‘industry’ or markets, but also involve a community or societal element. Sheltered independent housing should be seen from an integrated perspective. For a municipality, a proactive and management role should come to the fore. Ageing in place should also mean giving (potential) residents a participatory or initiating role in the decision-making process during the initiation phase. We have developed a couple of recommendations for sheltered independent housing, on the basis of which additional choices for portfolio diversification can be made. To that end, an atlas of sheltered independent living has been developed as an information and decision-making tool.

Now that we have presented and discussed the empirical findings regarding sheltered independent housing, we would like to conclude the article by critically reflecting on a very elementary discussion. Our current society favours as solution for an ageing society and for people in need the provision of special facilities. However, Petersen and Minnery (2013, p. 825) formulate a critical question that lies also behind the research presented here: ‘Why are there separate living spaces for older people?’ Or, going deeper along same lines: ‘How does specialised accommodation link to the social construction of old age?’ Both of these questions follow a line of arguments developed some time ago by Hugman (1999) and Laws (1993, 1994), discussing our specific relation with ageing from a critical academic point of view. Our findings provide a very differentiated picture of one element: sheltered independent housing. In our exploration of current practices regarding specialised accommodation, feeling at home, having the right mix of groups and social quality, the relation between public and private spheres is recurring issues. As was shown, scale and mix, the central elements of our research question, both matter. However, it has also been demonstrated that no clear optimum can be defined. We find variations in the provision of services, but almost always we also find a specific package that is offered in a distinct way and in a distinct place. No matter what we do or not, following from Petersen and Minnery (2013, p. 825; based on Laws 1993) argumentation, providing ‘specialised’ accommodation for older people is *inherently* ageist (and also relates to the need category). Our practice is that established between public authorities, be it health or social services, the health sector and the building industry of separating older people geographically and spatially. It has consequences for how society perceives older people, how they live and how they are viewed in that very same society. And in turn, the specific form of spatial separation affects the identity formation of older people. To critically reflect on this from, an academic point of view will help us create more appropriate spaces in the future.

**Fig. 11:**
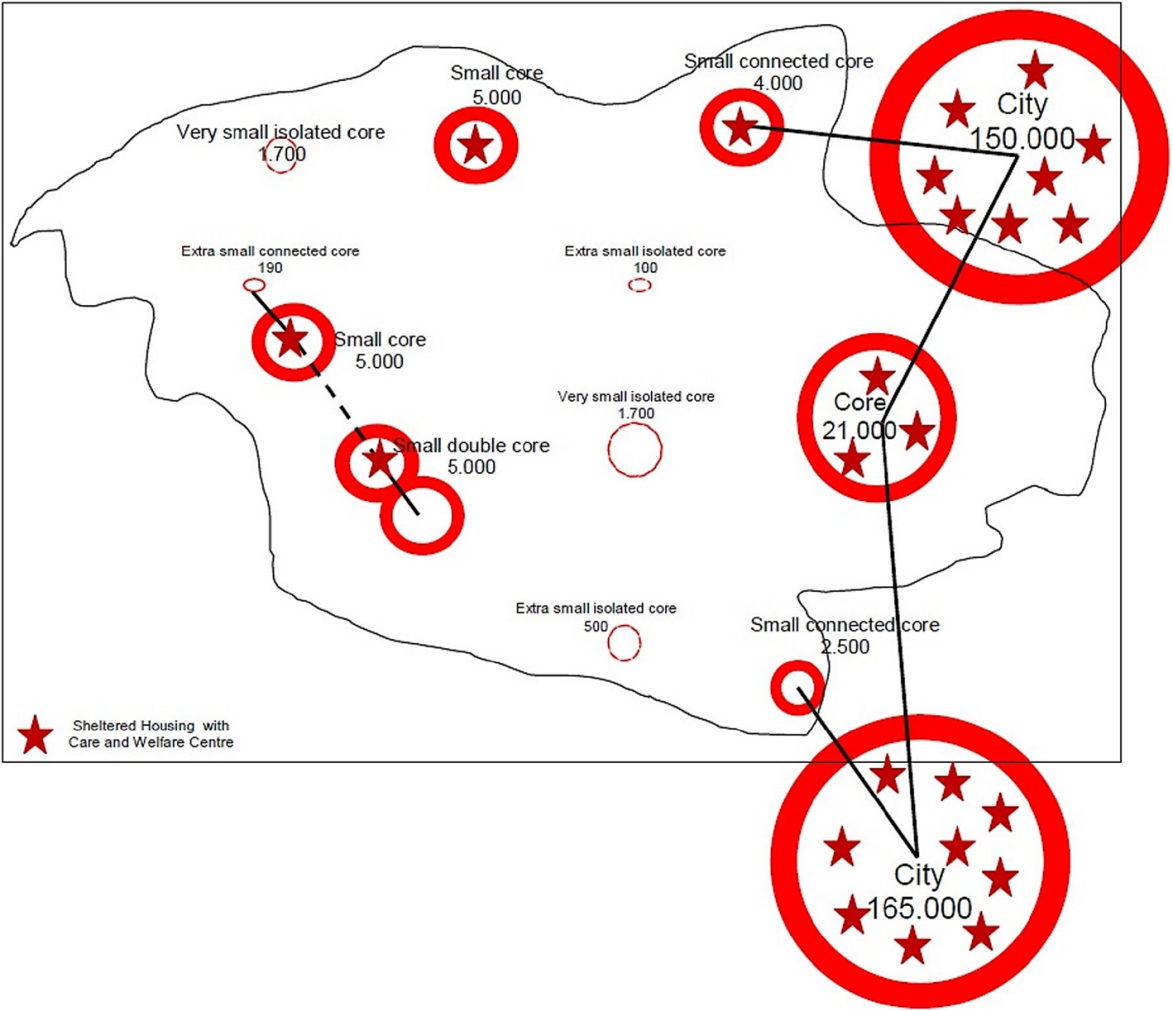
Functional region in a situation of ageing and declining

Are there alternatives? Will the new metropolis be multi-generational (EC and DG Regional Policy 2011, p. 40)? The issue at hand, also reflecting demographic developments and including different lifestyles, is adaptability, to which we will turn in our final point. According to the empirical results, an answer regarding the optimal scale cannot and does not need to be given. Relatively small, specialised accommodation complexes stand next to relatively large accommodation complexes that provide various services. The same applies to the range and variety of locations. A good mix of different groups and diversity of services seems to be the appropriate answer to the question: What are the preferred living conditions when a person is ageing OR in need? It seems that sheltered independent housing that is fit for the future depends on the quality and diversity of services provided, and it also depends on multi-locality, combinations of physical scale and location. In short, creating more opportunities to develop a wider range of projects and models becomes an issue (see Fig. 11 for a model situation in an existing region). In terms of required further research, our viewpoints should focus more on housing itself. We need to consider existing concepts for flexible and adaptable layouts of flats and ideas regarding multi-purpose design, on the level of individual buildings but also on the level of city quarters. Co-housing projects, especially multi- or intergenerational co-housing projects can be taken as a starting point (Ache and Fedrowitz 2012), researching options for a self-chosen construction of ageing in place.

### Postscript

The Sheltered independent Housing Atlas, www.bzwatlas.nl, provides an overview of all the research cases and a tool to test whether the physical scale and group mix of a newly built or existing sheltered independent housing project match the recommendations of this research.
